# A Quantum Expectation Value Based Language Model with Application to Question Answering

**DOI:** 10.3390/e22050533

**Published:** 2020-05-09

**Authors:** Qin Zhao, Chenguang Hou, Changjian Liu, Peng Zhang, Ruifeng Xu

**Affiliations:** 1 School of Computer Science and Technology, Harbin Institute of Technology, Shenzhen 518055, China; zhaoqin@hit.edu.cn (Q.Z.); cjliux@163.com (C.L.); 2 Center for Remote Imaging, Sensing and Processing, National University of Singapore, Singapore 119076, Singapore; a0086307@u.nus.edu; 3 School of Computer Science and Technology, Tianjin University, Tianjin 300050, China; pzhang@tju.edu.cn

**Keywords:** quantum language model, interpretability, quantum expectation value, density matrix, observable

## Abstract

Quantum-inspired language models have been introduced to Information Retrieval due to their transparency and interpretability. While exciting progresses have been made, current studies mainly investigate the relationship between density matrices of difference sentence subspaces of a semantic Hilbert space. The Hilbert space as a whole which has a unique density matrix is lack of exploration. In this paper, we propose a novel Quantum Expectation Value based Language Model (QEV-LM). A unique shared density matrix is constructed for the Semantic Hilbert Space. Words and sentences are viewed as different observables in this quantum model. Under this background, a matching score describing the similarity between a question-answer pair is naturally explained as the quantum expectation value of a joint question-answer observable. In addition to the theoretical soundness, experiment results on the TREC-QA and WIKIQA datasets demonstrate the computational efficiency of our proposed model with excellent performance and low time consumption.

## 1. Introduction

Recently, quantum inspired language models (LMs) have drawn increasing attention in Information Retrieval (IR) [1,2] and Natural Language Processing (NLP) tasks [3,4,5], for their excellent interpretability and comparable performance to strong Convolutional Neural Network (CNN) [6] and Long Short-term Memory (LSTM) [7] baselines. In contrast to classical LMs which utilize probabilistic models to measure the uncertainty of a text, quantum-inspired LMs are motivated by the quantum probability theory and can be considered as a generalization of the classical ones [8,9].

Sordoni, Nie and Bengio [4] for the first time proposed a Quantum Language Model (QLM) in IR. The probability uncertainties of words and word compounds are encoded in density matrices. Von-Neumann (VN) Divergence between question and answer density matrices are used to compute the matching score. As the first practical application of quantum probability, their model achieves substantial improvements over bag-of-words models.

Zhang et al. [5] proposed an end-to-end Neural Network based Quantum-like Language Model (NNQLM). Word embedding vectors as the analogy of state vectors, are applied to build question and answer density matrices. Then the combination of question and answer density matrices forms a joint representation which can measure the similarity between the question-answer pairs. Two different architectures are utilized to measure the joint representation, namely NNQLM-I and NNQLM-II. NNQLM-I extracts diagonal values and trace values, while NNQLM-II is built upon CNN.

In order to further explore the interpretability of neural networks, Li et al. [10] built a Complex-valued Network for Matching (CNM). Each word is encoded as a complex-valued vector, whose length represents the relative weight of the word, while the direction is considered as a superposition state. Local mixture scheme is adopted to construct a density matrix. A novel projection approach based on Gleason’s theorem is proposed to extract features from density matrices. Their model achieves comparable performances to CNN and RNN baselines.

Despite the exciting progress in quantum-inspired language models, there are still two challenges. The first challenge is to construct a unique density matrix shared among the word and sentence levels. In most QLMs, sentence is a semantic subspace in a quantum system and density matrix for each sentence semantic subspace is required to explored. However, in quantum theory, different statistical ensembles of pure states of the same Hilbert space can be described using the system’s unique density matrix. Represented as quantum states in the Hilbert Space, words and sentences should therefore be characterized by the unique density matrix, rather than composed of distinct ones. By preserving the consistence of density matrix, the total spatial degrees of freedom of the whole system can be diminished, which will result a more robust model. Secondly, only synthetic measures, lacking self-consistent quantum correspondence, have been employed in the aforementioned models, to evaluate the correlation coefficients between questions and answers. In particular, as an essential component of matching measurements in NNQLM-II, the convolutional layer over a joint representation for a question-answer pair is devoid of an analogous explanation in quantum probability theory. In addition, the projection measurement utilized in CNM also diminishes the interpretability from the perspective of quantum mechanism.

**Figure 1:**
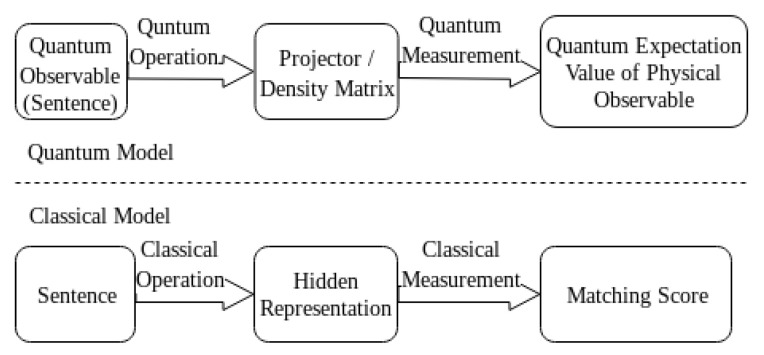
The correspondence between our quantum model and classical one.

To address the above two challenges, we come up with a Quantum Expectation Value based Language Model (QEV-LM), where a unique density matrix is constructed, and words and sentences are viewed as observables in the same quantum system. We firstly map each word as a complex-valued state in a Hilbert Space, followed by constructing observables for question and answer sentences. By direct multiplication, we build joint question-answer observables, whose quantum expectation value naturally dovetails with the matching score of question-answer pairs. Figure 1 illustrates the correspondence between our quantum model and the classical one. In addition to the model’s physical interpretability, it also exhibits higher computational efficiency with excellent performance and low time consumption.

Our main contributions can be summarized as follows:

- We propose QEV-LM, which represents words and sentences as different observables in a quantum system and utilizes a shared density matrix to measure joint question-answer observables. Under this scheme, the matching score of a question-answer pair is naturally explained as the quantum expectation value of the corresponding joint question-answer observable.
- We come up with a computationally efficient approach to constructing the shared density matrix via a quantum-like kernel trick.
- We apply QEV-LM to a typical answer selection Question Answering task on TREC-QA and WIKIQA datasets. Our model outperforms other quantum models with low time consumption and also surpasses strong CNN and LSTM baselines.
- A detailed discussion is conducted. In particular, we show that the off-diagonal elements of the density matrix, which correspond to sememes’ superpositions, play an important role to improve the model’s performance.

The rest of the paper is organized as follows. Section 2 presents a review of the related work, which motivates the proposed Quantum Expectation Value based Language Model as detailed in Section 4. Section 3 gives some relevant quantum physics terminology. Section 4 shows the detailed components of QEV-LM. Section 5 reports our experimental setup and results. The discussion is presented in Section 6. In Section 7, we conclude the paper and point out future research directions.

## 2. Related Work

In this section, we give a brief review of the related work on quantum-inspired work, including the recent work in Information Retrieval (IR) and and some representative work in Question Answering (QA).

Van Rijsbergen (2004) [3] for the first time proposed to adopt mathematical formalism to unify the logical, geometric and probabilistic IR models. Via the corresponding geometric representation in the Hilbert space of basic elements in IR, the traditional IR models are endowed with physical explanation. After this pioneering work, a number of quantum-inspired work [4,11,12,13] has been developed, based on the analogy between quantum phenomena and elements in natural language processing.

Inspired by the quantum double-slit experiment, Zuccon and Azzopardi [14] studied that the similarity between document ranking and quantum phenomena and proposed to consider texts’ interfere when calculating the relevance of different texts. Zhang et al. [11] modeled cognitive interference in the relevance judgement process, based on probabilistic automaton(PA) and quantum finite automaton. Piwowarski et al. [15] introduced information need vector space where events, such as document relevance or observed user interactions, correspond to subspaces.

Sordoni, Nie, and Bengio [4] proposed a principled Quantum Language Model (QLM), which generalizes the traditional statistical LM by adopting the probabilistic framework of quantum theory. This model is the first practical application quantum probability in language model. Density matrix is introduced to describe a more general representation for texts by looking at vector space model and language model in the quantum formalism. This model shows significant improvements over a robust bag-of-words baseline. Xie et al. [16] further improved QLM, by considering quantum entanglement.

Later, Zhang et al. [5] broadened work in IR to QA. An end-to-end quantum-like language model has been proposed. A new density matrix based on word embedding is designed to represent a sentence. Via matrix multiplication, question and answer density matrices can be combined to a joint representation, from where features can be extracted to measure the matching score. Experiment results show the effectiveness of the model. In order to give a better physical interpretability, Li et al. [10] constructed a complex-valued network, where words are encoded with complex-valued embedding, analogous to physical state in a quantum system. The proposed network for matching achieves comparable performances to typical CNN and RNN baselies.

Even though there are fruitful findings in quantum-inspired language models, current research are quite fragmented and facing two problems. First, in most studies of quantum language models, despite a whole quantum system with a complete Hilbert space is introduced, words and sentences usually are viewed as a sub event space, described by specific density matrices. The whole quantum system’s unique density matrix is lack of investigation. Second, in quantum-inspired models, only fragmented physical concept is integrated into models, without a systematical complete interpretability. For example, in NNQLM, words are endowed with physical state, but the later convolutional layer lacks of a corresponding physical explanation. In this paper, we aim to tackle the above two problems. We propose a Quantum Expectation Value based Language Model (QEV-LM). A unique density matrix is constructed which carries the whole information of the semantic Hilbert space. Words and sentences now are viewed as different observables in the same space. By calculating the expectation value of each observable using the unique density matrix, one can obtain the probability of each observable. Under this picture, the matching score of a question-answer pair is naturally explained as the quantum expectation of joint question-answer observable. The detailed components of QEV-LM are presented in Section 4.

## 3. Background

In this section, we briefly introduce some relevant quantum physics terminology.

### 3.1. Basic Concepts

In quantum probability theory [17], the probabilistic space is naturally represented in a vector space, specifically a Hilbert space, denoted as $H^{n}$. We use Dirac’s notation to denote a unit vector in this space. For example, a unit vector $\vec{u}\in H$ and its transpose ${\vec{u}}^{T}$ are respectively written as a *ket*|u〉 and a *bra*〈u|. The inner product between two state vectors is written as 〈u|v〉. The projector onto the direction |u〉 is an outer product of |u〉 itself, which is denoted as |u〉〈u|. Each rank-one projector |u〉〈u| represents a quantum elementary event, also called a dyad. After choosing the standard basis $\{|e_{j}\rangle \}\left(i=1,2,\cdots n\right)$ for sememes, any state vector |u〉 can be a superposition of the basis vectors:

(1) $$|u\rangle =\sum_{i=1}^{n}u_{i}|e_{i}\rangle .$$

where $u_{i}$ is the probability amplitude along $|e_{i}\rangle$ and satisfies $\sum_{i}u_{i}^{2}=1$.

A generalization of the conventional finite probability distributions in quantum probability theory is called density matrices [18]. A density matrix ρ can be defined as a mixture over dyads

(2) $$\rho =\sum_{i}p_{i}|\psi_{i}\rangle \langle \psi_{i}|,$$

where ${\left\{\psi_{i}\right\}}_{i=1}^{n}$ are pure states and $p_{i}\geq 0$ is the corresponding probability. Equivalently, the density matrix can be decomposed as

(3) $$\rho =\sum_{i}\lambda_{i}|k_{i}\rangle \langle k_{i}|,$$

where $\lambda_{i}$ is an eigenvalue, $|k_{i}\rangle$ is the corresponding eigenvector, and ρ is symmetric, positive sem-idefinite, and of trace 1. By Gleason’s theorem [19,20], every density matrix ρ uniquely corresponds to a quantum probability measure μ, according to

(4) $$\mu_{\rho }(|u\rangle \langle u|)=\mathrm{tr}(\rho |u\rangle \langle u|).$$

### 3.2. Quantum Expectation Value of an Observable

A projector is only one special type of observable in a quantum system. The quantum probability measurement can generalize to that of arbitrary observables. In quantum probability theory, an experimental setup is depicted by its observable to be measured and the state of the system. The expectation value of the observable O is the probabilistic expected value of the measurement [17,21]. That is, it is an average of all the possible outcomes of a measurement as weighted by their likelihood. Consider an observable O. In the commonly used case in quantum mechanics, ψ is a pure state in the Hilbert space. The expectation value is defined as 〈ψ|O|ψ〉. However, in system like thermodynamics and quantum optics, mixed states are of importance. The systems are described by their corresponding density matrix Equation (2), and the quantum expectation value of any observable O can be obtained as follows:

(5) $${\langle O\rangle }_{\rho }=\mathrm{tr}\left(\rho O\right)=\sum_{i}p_{i}\langle \psi_{i}\left|O\right|\psi_{i}\rangle .$$

when consider O=|u〉〈u|, Equation (5) is just Equation (4).

## 4. Quantum Expectation Value Based Language Model

**Figure 2:**
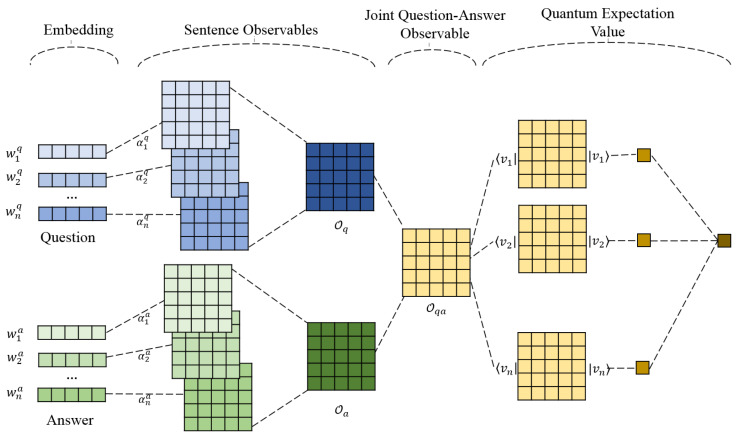
Quantum Expectation Value based Language Model (QEV-LM).

In quantum language model, there is a unique quantum system with a Hilbert space. All the physical event happens in this unique system. Under this background, our proposed Quantum Expectation Value Based Language Model (QEV-LM) is constructed, as shown in Figure 2. It consists of several parts: a word encoder, sentence observales, joint quenstion-answer observable and quantum expectation value as the matching score. Detailed explanation of each component is presented as follows.

### 4.1. Complex-Valued Word Embedding

Inspired by the fact that a quantum state is usually complex-valued, we naturally introduce the Semantic Hilbert Space $H^{n}$ on a complex vector space $C^{n}$. $H^{n}$ is spanned by a set of orthogonal basis states $\{|e_{j}\rangle {\}}_{j=1}^{n}$, with $|e_{j}\rangle$ being a sememe representing a semantic unit [22]. A unit state $|e_{j}\rangle$ is a one-hot vector, i.e., only the *j*-th element in $|e_{j}\rangle$ is one while all the other elements are zero.

A word *w* is treated as a superposition of sememes $\{|e_{j}\rangle {\}}_{j=1}^{n}$. Representing the Hilbert Space $H^{n}$ in a polar coordinate system, we can expand word *w* as follows:

(6) $$|w\rangle =\sum_{j=1}^{n}r_{j}e^{i\phi_{j}}|e_{j}\rangle ,$$

where $r_{j}$ is a non-negative real-value amplitude of the state |w〉 along the radius direction, satisfying $\sum_{j=1}^{n}r_{j}^{2}=1$, and $\phi_{j}\in \left[-\pi ,\pi \right]$ is the corresponding phase of the state |w〉 in the polar coordinate system.

Under the above constraints, we encode each word *w* as $\vec{w}$ with two sets of parameters. The first set consists of radius amplitudes, obtained from a word embedding lookup table $E\in R^{|V|\times d}$, where |V| is the length of the vocabulary and *d* is the dimension of the word embedding. The second set contains the corresponding phases, initialized with normally distributed random values between [−π,π]. We utilize a L2 normalization to restrict every word *w* to a unit length as follows:

(7) $$|w\rangle =\frac{\vec{w}}{\|\vec{w}\|},$$

where $\|\vec{w}\|$ denotes L2-norm of $\vec{w}$.

### 4.2. Sentence Level Observable Construction

For each single word $w_{i}$, the corresponding projector $\Pi_{i}=|w_{i}\rangle \langle w_{i}|$ is a observable to measure the probability of word $w_{i}$ in the Hilbert Space $H^{n}$ via Gleason’s theorem Equation (4), together with the density matrix of the system. Now, we try to construct an observable which can represent a sentence. Assuming that a sentence *s* has *n* words, with the corresponding dyads being $\{|w_{s1}\rangle \langle w_{s1}\left|,\right|w_{s2}\rangle \langle w_{s2}|,\cdots \}$, we claim that a sentence observable $O_{s}$ is obtained via

(8) $$\begin{array}{cc} Os & =\mathrm{MAX}(\alpha_{s1}|w_{s1}\rangle \langle w_{s1}|,\alpha_{s2}|w_{s2}\rangle \langle w_{s2}|,\cdots ), \end{array}$$

(9) $$\begin{array}{cc} \alpha_{si} & =\frac{\exp\left(\|{\vec{w}}_{si}{\|}_{p}\right)}{\sum_{j}\exp\left(\|{\vec{w}}_{sj}{\|}_{p}\right)}. \end{array}$$

Here word dyads have divergent contributions to the sentence representation, which are quantified by the weights $\alpha_{si}$, i.e., the soft-max normalization of the Lp-norm length $\|{\vec{w}}_{sj}{\|}_{p}$ of the word $w_{si}$ [23]. Then a max-pooling operation is performed to select the biggest items element-wisely along all the weighted dyads. The resultant sentence observable turns out to be excellent in representing the sentence features. This is one of the novelties of this paper. Instead of building a sentence density matrix which follows a standard procedure, the way to design a sentence-level observable is more flexible. This gives us a chance to find a more powerful representation.

### 4.3. Joint Question-Answer Observable

After performing the above operations on a pair of question and answer sentences, we can obtain the question observable $O_{q}$ and answer observable $O_{a}$, respectively. Usually a distance-based score like cosine similarity is computed to measure the similarity between a question-answer pair. Here, inspired by Hu et al. [24]’s and Wang et al. [25]’s work where a joint representation used in the matching model has been proven to be effective, we build a joint question-answer observable $O_{qa}$ via element-wise multiplication as follows:

(10) $$O_{qa}=O_{q}O_{a}.$$

Compared to matrix multiplication which mixes elements in the same row and column, the element-wise multiplication focuses on the similarity of the corresponding elements of question and answer observables via straight multiplication. From mathematical point of view, for element-wise multiplication, back propagation can improve the element representation more straightforward and efficiently. This can give us a better joint question-answer representation. In next subsection, we will show that the matching score in our quantum system is just the quantum expectation value of the joint question-answer observable $O_{qa}$.

### 4.4. Quantum Expectation Value of the Joint Question-Answer Observable

Remember that we introduce the same Sematic Hilbert Space $H^{n}$ (Section 4.2) for all word and sentence observables. Instead of considering respective density matrices for question and answer sentences, it is more reasonable to find the shared density matrix underlying the Semantic Hilbert Space. Then the quantum expectation value for any arbitrary observable O can be calculated according to Equation (5).

So the essential task is to seek the desired density matrix. However, either to check the pure states $\{|\psi_{i}\rangle {\}}_{i=1}^{n}$ or to keep the orthogonal basis $\{|k_{i}\rangle {\}}_{i=1}^{n}$ as shown in Equations (2) and (3) is at the cost of tremendous computation, which would be a disaster for the training process. To overcome this difficulty, we learn from the *kernel trick* in traditional machine learning with a kernel function $k\left(x,x^{\left(i\right)}\right)=\phi \left(x\right)\cdot \phi \left(x^{\left(i\right)}\right)$ [26]. Instead of finding the explicit form of ϕ(x), a kernel function *k* often admits an implementation that is significantly more computationally efficient. Similarly, in our case, instead of finding the explicit standard orthogonal basis $\{|k_{i}\rangle {\}}_{i=1}^{n}$ or pure states $\{|\psi_{i}\rangle {\}}_{i=1}^{n}$, we only focus on the construction of the final density matrix, which is *symmetric*, *positive sem-idefinite*, and *of trace* 1. So this density matrix is a quantum-like kernel function. We choose the general form of the system’s density matrix being:

(11) $$\rho =\sum_{i=1}^{m}|v_{i}\rangle \langle v_{i}|,$$

where *m* is the total number of states $|v_{i}\rangle$ to form ρ, and ${\left\{v_{i}\right\}}_{i=1}^{m}$ are unknown *n*-dimension vectors to be trained. Note that *m* does not need to be equal to the dimension *n* of the Hilbert Space and $\{|v_{i}\rangle {\}}_{i=1}^{m}$ can be any arbitrary vectors. Now, we prove that Equation (11) is an allowable density matrix which is *symmetric*, *positive sem-idefinite*, and *of trace* 1.

- Be symmetric. First of all, $|v_{i}\rangle \langle v_{i}|$ for every $v_{i}$ is symmetric. $|v_{i}\rangle \langle v_{i}{|}_{ab}=|v_{i}\rangle \langle v_{i}{|}_{ba}$, for i∈{1,2,⋯,m}. Therefore
  $$\begin{array}{c} \rho_{ab}=\sum_{i=1}^{m}|v_{i}\rangle \langle v_{i}{|}_{ab}=\sum_{i=1}^{m}|v_{i}\rangle \langle v_{i}{|}_{ba}=\rho_{ba}. \end{array}$$
- Be semi-definite. A matrix *M* (rank *n*) is said to be semi-definite if 〈z|M|z〉 is positive or zero for every non-zero column vector *z* of *n* numbers [27]. We can show that
  $$\begin{array}{cc} \langle z|\rho |z\rangle & =\langle z|(\sum_{i=1}^{m}|v_{i}\rangle \langle v_{i}|)|z\rangle \\ & =\sum_{i=1}^{m}\langle z|v_{i}\rangle \langle v_{i}|z\rangle =\sum_{i=1}^{m}{\langle v_{i}|z\rangle }^{2}\geq 0. \end{array}$$
- Be of trace 1. After the density matrix is constructed, one can always multiply a scalar to the matrix to make its trace being 1 without violating the symmetric and semi-definite properties. Since this scalar can be shifted to the later pipeline treated as a rescale operation to our parameters, we do not directly restrict the trace of density matrix in our model.

Given the shared density matrix Equation (11), according to Equation (5), we can compute the quantum expectation value of the joint question-answer observable $O_{qa}$, which is:

(12) $${\langle O_{qa}\rangle }_{\rho }=\mathrm{tr}\left(\rho O_{qa}\right)=\sum_{i=1}^{m}\langle v_{i}|O_{qa}|v_{i}\rangle .$$

Since the joint question-answer observable depicts the similarity between question and answer sentences, it is natural to link it to the matching score of a question-answer pair. *Therefore, we find a new explanation for the matching score which is the quantum expectation value of the joint question-answer observable $O_{qa}$.*

### 4.5. Leaning to Rank

The probability of the positive label is viewed as the matching score for ranking. The negative cross entropy loss is used to train the back propagation:

(13) $$L=-\sum_{i}^{N}\left[y_{i}\log\left({\langle O_{qa}\rangle }_{\rho }\right)+\left(1-y_{i}\right)\log\left(1-{\langle O_{qa}\rangle }_{\rho }\right)\right].$$

## 5. Experiments

### 5.1. Experimental Setup

The experiments are conducted on two widely used benchmarking datasets for the Question Answering (QA) task, summarized in Table 1.

- TREC-QA [28] is a standard QA dataset in the Text REtrieval Conference (TREC).
- WIKIQA [29] is an open domain QA dataset released by Microsoft Research.

For both datasets, the task aims to select the most suitable answer for a question. Before our training process, data cleaning process is operated to make sure that every question has at least one correct answer. The evaluation metrics used to measure the performance of models are two commonly used rank-based metrics for the same task with the same datasets, namely Mean Average Precision (MAP) and Mean Reciprocal Rank (MRR).

### 5.2. Baselines for Comparison

A comprehensive comparison with a wide range of models is made. Since QEV-LM is quantum inspired, it is natural and necessary to compare with other closed quantum inspired models. They include

- **QLM** [4]. Density matrices $\rho_{q}$ and $\rho_{a}$ are used to represent question and answer sentences, respectively. Von-Neumann (VN) Divergence between $\rho_{q}$ and $\rho_{a}$ is used to measure the matching score between question and answer pairs [18].
- **NNQLM-II** [5]. It is an end-to-end language model. Embedding vector is introduced to encode question and answer sentences. The matching score is computed over the joint representation of question and answer density matrices.
- **CNM** [10]. It is a complex-valued matching network. Sentence is modeled with local mixture density matrices. Projectors select features for question and answer density matrices, and cosine similarity is used to calculate the matching score.

In addition, we also pick a couple of basic and typical CNN-based or LSTM-based QA models for comparison. They include Ngram-CNN [30,31], Multi-Perspective CNN (MP-CNN) [32], Long Short-term Memory with attention (LSTM-attn) [33], three-layer stacked bidirectional Long Short-term Memory with BM25 (Three-Layer BLSTM + BM25) [34]. It also should be mentioned that since quantum language models aims to find a more fundamental physical explanation for language models, we only chose the basic neural network for comparison as other papers on QLMs. The main baselines for comparison are those quantum-inspired models.

### 5.3. Implementation Details

QEV-LM is implemented by PyTorch [35]. The trainable parameters are the amplitudes ${\left\{r_{wi}\right\}}_{i=1}^{n}$ for each word *w*, the corresponding phases ${\left\{\phi_{wi}\right\}}_{i=1}^{n}$, and the vectors $\{|v_{i}\rangle {\}}_{i=1}^{m}$ that contribute to the shared density matrix. ${\left\{r_{wi}\right\}}_{i=1}^{n}$ are initialized with 50-dimension Glove vectors and ${\left\{\phi_{wi}\right\}}_{i=1}^{n}$ are initialized with random uniform distributed variables between [−π,π]. $\{|v_{i}\rangle {\}}_{i=1}^{m}$ are initialized with orthogonal complex-valued vectors. We adopt the Adam optimizer with the learning rate among [1e−4,5e−4,1e−3]. The batch size is tuned around [16,32,64]. L2 regularization is performed for the amplitudes ${\left\{r_{wi}\right\}}_{i=1}^{n}$ with a coefficient amid [5e−7,5e−6,5e−5]. We train our model for 100 epochs and the best model obtained in the dev dataset is used to evaluate in the test dataset.

### 5.4. Experimental Results

As shown in Table 2, our model wins 3 best performances out of the 4 metrics on TREC-QA and WIKIQA. The detailed comparison between the results of our model and those of other baselines is presented as follows:

First, in the scope of quantum language models, a) on TREC-QA dataset, QEV-LM significantly outperforms QLM by 19.49% on MAP and 22.97% on MRR, respectively; it exceeds NNQLM-II by 6.81% on MAP and 8.24% on MRR, respectively; it also surpasses CNM by 5.26% on MAP and 3.99% on MRR, respectively; b) On WIKIQA dataset, QEV-LM outperforms QLM significantly by 32.08% on MAP and 34.97% on MRR, respectively; it performs better than NNQLM-II with a rate of 3.88% on MAP and 5.37% on MRR, respectively. The result of our model is comparable with that of CNM on MAP and better than that of CNM on MRR with a rate of 1.22%. The improvement on TREC dataset is more manifest than that on WIKIQA. We find that the average length of question sentence of TREC-QA is much more closer to that of answer sentence, compared with the situation in WIKIQA. This can benefit the representation of joint question-answer observable, and hence the final performance.

Second, compared with typical CNN-based or LSTM-based QA models, our model shows better performance on all MAP and MRR measurements. This manifest that the two dimensional sentence observable in QEV-LM can learn more semantic interference than sentence vector representation in traditional language models.

## 6. Discussion

### 6.1. Ablation Study

We conduct an ablation analysis to investigate the influence of each component on our proposed model. The ablation studies are divided into three groups to investigate the respective effects of the Hilbert Space, observables and the shared density matrix. Experiment results are shown in Table 3 and explained as detailed below:

#### 6.1.1. Ablation Study on Hilbert Space

Remember that the Semantic Hilbert Space $H^{n}$ is a complex vector space $C^{n}$. Each state in this quantum system is encoded with a complex-valued vector whose amplitude part corresponds to the classical word embedding and phase part carries additional semantic information. Here, we examine the contribution of the complex-valued setup by reducing the Hilbert Space $H^{n}$ to a real vector space. In this space, QEV-LM-real is built with word vectors and the shared density matrix replaced by their real counterparts. On TREC-QA dataset, Table 3 shows that QEV-LM-real is 0.05% and 2.97% lower than QEV-LM on MAP and MRR, respectively. On WIKIQA dataset, QEV-LM-real is lower than QEV-LM by 3.53% on MAP and 4.42% on MRR. Therefore, the imaginary part carrying additional semantic information can improve the model’s performance. By the way, Table 2 and Table 3 illustrate that QEV-LM-real, with the influence of imaginary parts eliminated, also outperforms NNQLM-II (a QLM with real embeddings), which demonstrates the architecture’s superiority of QEV-LM.

#### 6.1.2. Ablation Study on Observables

Formerly, sentence observables $O_{s}$ are constructed via an element-wise maxpooling over a set of weighted word projectors $\{|w_{i}\rangle \langle w_{i}|\}$. But a variety of sentence observables can be designed via other flexible ways, e.g., directly maxpooling over word projectors without weights, which generates a model named as QEV-LM-no-weight. Table 3 shows that all results of QEV-LM-no-weight are quite close to those of QEV-LM, meaning that the weights are fine-tune parameters. However, when using the sentence observables with word projectors summed, on TREC-QA dataset, the results of QEV-LM-sum drop dramatically from those of QEV-LM by 18.22% on MAP and 17.02% on MRR; on WIKIQA dataset, there is also a big jump on both MAP and MRR. Even though the more important words contribute more to the sentence observable, after accumulation in summation irrelevant words can introduce a lot noise. Especially, compared to traditional sentence vector representation, observable is a two-dimensional matrix which can be ruined for more elements carrying noise. It is noted that our maximization operation is an element-wise operation over all word projectors, so the sentence observable carries the most important information of all words. This ablation study show than s that a suitable observable plays a vital role in feature extraction, similar to the cases of classical language models where better feature selection would produce higher model performance.

#### 6.1.3. Ablation Study on Density Matrix

**Figure 3:**
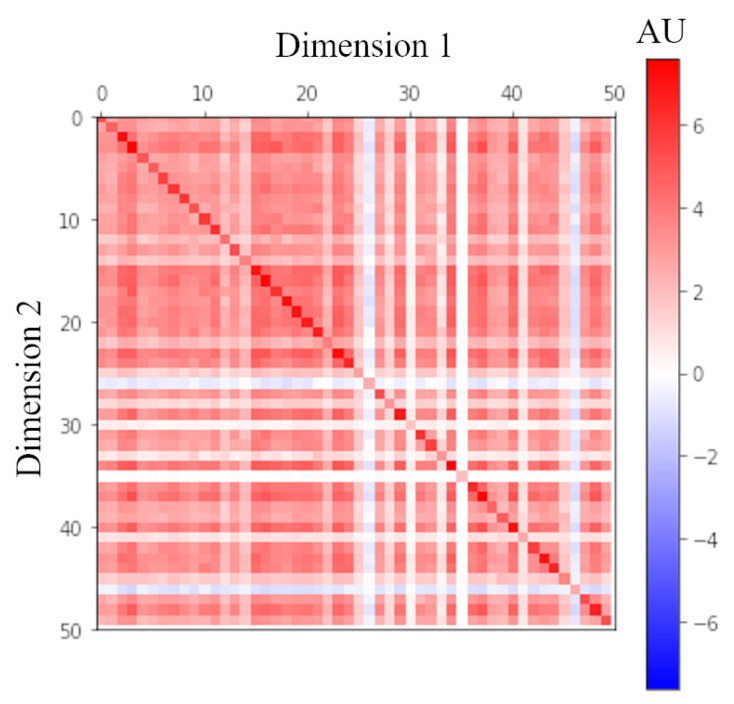
Real part of the density matrix $\rho =\sum_{i=1}^{m}|v_{i}\rangle \langle v_{i}|$. This is a 50×50 matrix due to that real $|v_{i}\rangle$ is a 50 dimensional vector. According to the color bar in the right, the value of each element of density matrix can be found.

The general form of a density matrix is $\rho =\sum_{i=1}^{n}p_{i}|\psi_{i}\rangle \langle \psi_{i}|$. In our QEV-LM, to avoid tremendous calculation, a quantum-like kernel trick is used to built the density matrix. However, when $\{|\psi_{i}\rangle {\}}_{i=1}^{n}$ are one-hot orthogonal basis, the density matrix reduces to a diagonal matrix with zero-valued off-diagonal elements, and this matrix corresponds to the probabilities of sememes in a classical case without sememes’ superpositions. To explore the contribution of the superpositions in the density matrix, two comparative models are built, namely QEV-LM-class1 and QEV-LM-class2. Within QEV-LM-class1, the density matrix is diagonal and contains only 2n parameters instead of m×2n. (In QEV-LM, the density matrix $\rho =\sum_{i=1}^{m}|v_{i}\rangle \langle v_{i}|$ is formed by m× complex-valued *n*-dimensional vectors.) In order to keep the same number of parameters, we build QEV-LM-class2 with each of the density matrix’s *n* diagonal elements being the summation of other *m* parameters. Table 3 shows that the performance of QEV-LM-class1 and QEV-LM-class2 drops dramatically, which demonstrates the effectiveness of the superpositions. After the training process, all parameters ${\left\{v_{i}\right\}}_{i=1}^{m}$ can be used to recover the density matrix as shown in Figure 3. It is reasonable that the contribution from diagonal elements corresponding to the classical probabilities is dominant, but the off-diagonal elements’ values are not neglectable. In conclusion, the sememes’ superpositions contribute substantially to the model’s performance.

### 6.2. Parameter Scale and Time Consumption

As shown in Table 4, our proposed QEV-LM reveals a comparable number of parameters as CNM [10] and in NNQLM-II [5]. Contributed from the word embedding and the shared density matrix, QEV-LM possesses |V|×2n+m×2n parameters, with |V|, *n*, and *m* as the vocabulary size, the embedding dimension, and the number of vectors ${\left\{v_{i}\right\}}_{i=1}^{m}$ respectively. Due to |V|≫m in normal cases, the scale of word embedding is dominant, which yields a similar model size as NNQLM-II and CNM. The source codes of aforementioned two models are provided in Theano and Keras, respectively. In order to exclude the effects of different machine learning libraries, we reimplement both of them in PyTorch to benchmark their inference speeds. We choose QEV-LM-real as the counterpart of NNQLM-II, since the latter only considers real word embedding.

Moreover, due to our straightforward approach to constructing the observables and the shared density matrix, our model is more lightweight and computationally efficient. Table 4 characterizes different models in our performance evaluation, which is conducted on a single GTX 1080 GPU by measuring the average inference time of a 256-sample batch. Our QEV-LM model achieves up to 17.2× speedup over CNM on the same platform, without sacrificing speed significantly compared with NNQLM-II, while QEV-LM-real with real embedding vectors achieves the highest speed over the others.

Therefore, *we conclude that besides the physical interpretability, our model is computationally efficient with excellent performance and low time consumption.* This proves the effectiveness of observables and the shared density matrix which make the model more flexible and robust.

### 6.3. Comparison on Physical Interpretability

One of the main motivation to introduce quantum language models is their powerful physical interpretability. In this subsection, we analyze the comparison of the explanation on each component of our model with that of other models, as shown in Table 5. On the word encoder layer, QEV-LM and CNM encode words as complex-valued physical states, which are more similar to a real physical state in a quantum system. As for sentence representation, in many QLMs, a sentence is an information subspace of the quantum system, characterized by a specific density matrix. In those cases, the whole quantum system is divided into many subspaces, and the system itself which has a unique density matrix carrying a complete semantic information is lack of utilization. The similarity between different density matrices of subspaces is analyzed using mathematical methods such as VN-divergence. Especially, the convolutional layer used to extract density matrix’s features in NNQLM-II is devoid of an analogous explanation in quantum probability theory. In our model, the whole quantum Hilbert space is not divided, and sentences are represented as quantum observables via word projector operators in the whole quantum system. Based on question’s and answer’s representation, a joint observable $O_{qa}$ can be constructed for each question-answer pair. Then, the unique density matrix of the system can be utilized to measure the probability, i.e., quantum expectation value, of this joint observable. Therefore, we end up with the physical interpretation that the matching score can be viewed as the quantum expectation value of the joint question-answer pair observable.

## 7. Conclusions and Future Work

In this paper, we propose a Quantum Expectation Value based Language Model (QEV-LM), with a shared density matrix constructed via a quantum-like kernel trick. This shared density matrix is the semantic Hilbert space’s unique density matrix. Within this framework, words and sentences are treated as quantum observables in the Hilbert Space. The question-answer matching score is naturally explained as the quantum expectation value of the joint question-answer observable. We apply our model on a typical answer selection task on standard benchmarking datasets, namely TREC-QA and WIKIQA. Experiment results on those datasets demonstrate the effectiveness of our proposed QEV-LM. Our model surpasses basic and typical CNN and LSTM baselines on both datasets and especially outperforms other quantum-inspired LMs with low time consumption. In conclusion, our model is not only advantageous with its physical interpretability, but also practically well-performed.

Our ablation studies show that different observables can dramatically affect the model’s performance, and the off-diagonal elements corresponding the semantic superpositions significantly boost the model’s performance. Therefore, it would be interesting to further explore the possible observables and other approaches to construct superpositions [36].

## Figures and Tables

**Table 1:** Statistics of TREC-QA and WIKIQA Datasets.

Dataset		Train	Dev	Test
TREC-QA	Questions	1229	65	68
	Pairs	53,417	117	1442
WIKIQA	Questions	837	126	633
	Pairs	8627	1130	2351

**Table 2:** Results on TREC-QA and WIKIQA. The best performed values are in bold.

Model	TREC-QA		WIKIQA	
	MAP	MRR	MAP	MRR
Ngram-CNN	0.6709	0.7280	0.6661	0.6851
MP-CNN	0.7770	0.8360	/	/
Three-Layer BLSTM + BM25	0.7134	0.7913	/	/
LSTM-attn	/	/	0.6639	0.6828
QLM	0.6784	0.7265	0.5109	0.5148
NNQLM-II	0.7589	0.8254	0.6496	0.6594
CNM	0.7701	0.8591	0.6748	0.6864
QEV-LM	0.8106	0.8934	0.6748	0.6948
Over CNM	5.26%	3.99%	0%	1.22%

**Table 3:** Ablation analysis.

Model	TREC-QA		WIKIQA	
	MAP	MRR	MAP	MRR
QEV-LM-real	0.8066	0.8669	0.6510	0.6641
QEV-LM-no-weight	0.8049	0.8895	0.6729	0.6934
QEV-LM-sum	0.6629	0.7413	0.6226	0.6406
QEV-LM-class1	0.7391	0.8053	0.6007	0.6182
QEV-LM-class2	0.7402	0.8023	0.6049	0.6195
QEV-LM	0.8106	0.8934	0.6748	0.6948

**Table 4:** The comparison on Parameters (Params) and Time Consumption (TC). Here, time consumption is the average inference time for a batch of 256 samples.

Model	Params	TC
NNQLM-II	3.03 M	53.2 ms
QEV-LM-real	2.97 M	45.0 ms
CNM	5.90 M	1.50 s
QEV-LM	5.89 M	87.5 ms

**Table 5:** Comparison on physical explanations of different QLMs.

Component	Model	Physical Explanation
**Word Encoder**	QLM	one-hot embedding
	NNQLM-II	real-valued physical state
	CNM	complex-valued physical state
	QEV-LM	complex-valued physical state
**Sentence representation**	QLM	Sentence density matrix
	NNQLM-II	Sentence density matrix
	CNM	Sentence density matrix via local density matrices scheme
	QEV-LM	Sentence Observable via word project operators
**Question-Answer pair**	QLM	question density matrix $\rho_{q}$ and answer density matrix $\rho_{a}$
	NNQLM-II	joint question-answer density matrix $\rho_{qa}$
	CNM	question density matrix $\rho_{q}$ answer density matrix $\rho_{a}$
	QEV-LM	joint question-answer observable $O_{qa}$
**Matching score**	QLM	VN-divergence
	NNQLM-II	softmax valud of CNN-based density features
	CNM	Cosine distance of density features
	QEV-LM	Quantum expectation value of joint observable
